# Red Flags and Adversities on the Way to the Robust CE-ICP-MS/MS Quantitative Monitoring of Self-Synthesized Magnetic Iron Oxide(II, III)-Based Nanoparticle Interactions with Human Serum Proteins

**DOI:** 10.3390/molecules27238442

**Published:** 2022-12-02

**Authors:** Jacek Sikorski, Marcin Drozd, Magdalena Matczuk

**Affiliations:** 1 Chair of Analytical Chemistry, Faculty of Chemistry, Warsaw University of Technology, Noakowskiego St. 3, 00-664 Warsaw, Poland; 2 Chair of Medical Biotechnology, Faculty of Chemistry, Warsaw University of Technology, Noakowskiego St. 3, 00-664 Warsaw, Poland; 3 Centre for Advanced Materials and Technologies CEZAMAT, Warsaw University of Technology, Poleczki St. 19, 02-822 Warsaw, Poland

**Keywords:** superparamagnetic iron oxide nanoparticles, capillary electrophoresis, inductively coupled plasma tandem mass spectrometry, albumin, transferrin

## Abstract

The growing interest in superparamagnetic iron oxide nanoparticles (SPIONs) as potential theranostic agents is related to their unique properties and the broad range of possibilities for their surface functionalization. However, despite the rapidly expanding list of novel SPIONs with potential biomedical applications, there is still a lack of methodologies that would allow in-depth investigation of the interactions of those nanoparticles with biological compounds in human serum. Herein, we present attempts to employ capillary electrophoresis-inductively coupled plasma tandem mass spectrometry (CE-ICP-MS/MS) for this purpose and various obstacles and limitations noticed during the research. The CE and ICP-MS/MS parameters were optimized, and the developed method was used to study the interactions of two different proteins (albumin and transferrin) with various synthesized SPIONs. While the satisfactory resolution between proteins was obtained and the method was applied to examine individual reagents, it was revealed that the conjugates formed during the incubation of the proteins with SPIONs were not stable under the conditions of electrophoretic separation.

## 1. Introduction

Despite the COVID-19 outbreak in late 2019, cancer is still the second leading cause of death worldwide and a significant problem in public health care [1,2]. Traditional cancer therapies such as chemotherapy or radiation therapy have been associated with adverse side effects. In order to limit non-selectivity toward cancer cells and thus systemic toxicity, new strategies are sought. Of great interest are nanomaterials and their use as potential theranostic agents [3]. Cancer theranostic is a relatively new medical approach that combines diagnosis and therapy to remove a solid tumor in a non-invasive way. Integration of those elements in a single nanoplatform allows for real-time monitoring of treatment progress and efficiency [4].

There are many nanomaterials with the potential to be used in cancer treatment, and among them, superparamagnetic iron oxide nanoparticles (SPIONs) are particularly noteworthy due to their properties. They consist of iron oxide in the form of magnetite (Fe_3_O_4_). As a consequence of their size being below 20–30 nm (with only one magnetic domain within one nanoparticle), they exhibit superparamagnetic properties: they can reversibly change their magnetic moment after exposure to the external magnetic field [5,6]. Because of their low cytotoxicity, biocompatibility, stability, and unique magnetic properties, they are extensively researched for theranostic purposes. Their possible applications include magnetic resonance imaging (as contrast agents), photothermal therapy, magnetic fluid hyperthermia, or radiotherapy [7]. Moreover, they can be easily tailored in size, morphology, and surface functionalization to give them appropriate application fields, e.g., as chemo- or immunotherapeutic carriers [8].

Many examples of novel SPIONs with potential theranostic properties are presented in the literature [9]. A variety of techniques thoroughly characterizes the newly designed NP, e.g., X-ray diffraction (XRD), dynamic light scattering (DLS), Fourier transform infrared spectroscopy (FTIR), mass spectrometry (MS), scanning electron microscopy (SEM) or transmission electron microscopy (TEM) [10,11,12]. Unfortunately, an important aspect is usually omitted during that examination. What happens with those SPIONs after introduction into biological media? The issue was recently discussed in detail by Timerbaev A.R. in the review paper [13]. Namely, after contact with human blood, SPIONs undergo biochemical transformations—proteins in the serum form the corona around NPs [14]. Various surface modifications can be used to adjust the corona composition and, as a result, determine the behavior of SPIONs in vivo [15,16]. However, the scarcity of appropriate methods that could be used to study that behavior is a major obstacle to developing novel SPIONs for biomedical applications.

There are three aspects regarding the interactions of SPIONs with biological compounds that should be examined before their implementation into clinical use. First, what proteins are building the corona? The proteomic analysis comes in handy here, allowing for their identification employing mass spectrometry techniques [16,17,18]. Second, what SPION alterations take place in biological media? Only a few studies can be found in this matter, which explores the NP’s stability after contact with human serum proteins. Namely, by application of DLS or single particle ICP-MS/MS (spICP-MS/MS), the size distribution changes (related to the agglomeration level of SPIONs) were examined after incubation with proteins [19,20]. Furthermore, sector field ICP-MS was employed to monitor the degradation of iron oxide NPs in the samples with human serum [21]. The third aspect is related to the kinetics of those biotransformations and is the least explored due to the complexity of interactions and lack of proper methodologies. Researchers have attempted to examine this issue, e.g., by employing ICP-MS to monitor the changes in the content of the particular metals (Cu, Mn) present in the metalloproteins building corona [22]. However, this method is limited in its applicability and cannot be used for an in-depth quantitative investigation of those interactions. To summarize, while there are various means to identify proteins building the corona, there is still a lack of quantification methodologies that could be used to investigate protein–SPION conjugates and protein ligands’ binding kinetics. Moreover, there is a need for tools allowing the separation and detection of different nano–biospecies forming in serum samples—most of the research currently focuses on interactions between NPs and single proteins, omitting the crucial matter of competition between compounds forming the corona in the blood environment.

Hyphenated analytical techniques can be applied to study the alterations of SPIONs in human serum and resultant conjugates. ICP-MS/MS is an obvious choice for detection due to its high sensitivity to metal determination and ability to reduce polyatomic spectral interferences common during iron analysis. Among separation techniques commonly coupled with ICP-MS/MS, capillary electrophoresis appears to be a cutting-edge alternative to high-pressure liquid chromatography (HPLC) due to lower sample consumption and higher resolution. There are a few examples of CE applications to determine differently modified SPIONs [23,24,25], but in the case of biological matrices and human serum samples, mainly other nanomaterials have been tested till now [26,27,28].

Our previous study [29] presented the CE-ICP-MS/MS method for determining SPIONs under physiological conditions. The application of triple quadrupole and the oxygen used as collision/reaction gas allowed for simultaneous analysis of iron (^56^Fe^16^O^+^, marker of SPIONs) and sulfur (^32^S^16^O^+^, marker of proteins) and also minimized interferences occurring for iron. The optimized method was primarily used to investigate commercially available differently charged SPIONs. In addition, simple tests were also carried out to examine the method’s applicability in matrices containing proteins, namely albumin. However, the optimized ICP-MS/MS methodology can be effectively used to monitor signals from both SPIONs and human serum proteins, but the separation method was insufficient to observe the synthesized nanoparticle (NP) changes in the presence of whole human serum. The main limitation was the poor resolution of signals.

For the abovementioned reasons, we decided to develop the novel CE-ICP-MS/MS method in the frame of this study. During the optimization process, the emphasis was placed on improving the method resolution to obtain acceptable separation of signals from the two most abundant human serum transporting proteins—albumin and transferrin. The optimized methodology was then used to effectively portray the interactions of self-synthesized SPIONs with single serum proteins. Why self-synthesized? While their quality and size distribution does not always match that of the commercially available SPIONS, this study aimed to provide a basis for methodologies that could be used to investigate newly designed nanoparticles with various surface modifications and their interactions with biological compounds in laboratory environments. This work concerns the successive stages of research, and multiple problems noticed along the investigation pathway.

## 2. Results and Discussion

### 2.1. ICP-MS/MS Detection Method Optimization

A previously elaborated ICP-MS/MS methodology was used as a basis for the current research, and the original method can be found in the previous study [29]. Some of the detection parameters were marginally changed in order to obtain higher signal intensity. Moreover, additional instrument tuning has been performed with more detailed optimization of the collision/reaction gas flow rate. The main values based on which specific parameter was chosen were the intensity of signals and the signal-to-noise ratio for the sulfur (^32^S^16^O^+^) and iron (^56^Fe^16^O^+^) signals. The best results were obtained for 30% of oxygen at the maximum flow rate (0.45 mL/min). The effect of evaluating the oxygen flow rate in the collision/reaction cell (CRC) is presented in ESM Figure S1, and the final operational parameters are summarized in Table 1.

### 2.2. CE Separation Method Optimization

The main objective of the CE method optimization was to obtain the highest separation efficiency and resolution between the signals corresponding to specified reagents and products. Without distinctively separated signals corresponding to proteins, it would be impossible to clearly determine the composition of formed conjugates in a mixture of SPIONs and proteins. The separation conditions were optimized using two samples: one with albumin and transferrin (1 mg/mL each) and the other containing commercially available SPIONs with carboxyl-functionalized groups (Fe_3_O_4_@COOH, 15 µg/mL Fe), all diluted with incubation buffer.

Various combinations were investigated throughout the research, e.g., different background electrolyte (BGE) configurations, sample stacking techniques, or elements of isotachophoretic separation. Among those, Tris hydrochloride as the BGE in standard CE mode generated the best results. While the good quality of the signals (shape, width at the base) was desired, the choice was made based on the resolution between proteins and the stability of the interface—reagents that could cause aspiration problems, in the long run, had to be discarded. Next, three different concentrations of Tris hydrochloride buffer (containing 5, 10, and 20 mM of Tris hydrochloride and pH adjusted to 7.4 using NaOH) were tested, but it was noted that an increase in this parameter was associated with significant deterioration in the quality of signals and an unstable baseline. For this reason, 5 mM Tris hydrochloride was selected as the BGE. The addition of the NaOH solution was used then to adjust the pH to 7.4 and simulate the physiological environment. For the same reason, the capillary temperature was set to 37 °C during all analyses.

In the next step, the effect of the sample injection volume was examined. As expected, the reduction in the sample loading resulted in the improvement of albumin and transferrin separation factors (ESM, Figure S2). The hydrodynamic injection of 20 mbar per 5 s was chosen for further analyses. Afterward, the voltage applied to the capillary was selected. Lowering the voltage, despite significantly improving the separation of protein signals in relation to SPIONs, was also associated with reducing the signals’ qualities. An increase in peak width resulted in a lower resolution between albumin and transferrin. The voltage of +15 kV was chosen to compromise the quality of monitored signals and the separation between proteins (ESM, Figure S3).

The optimized parameters are presented in Table 2, while the electropherograms for the separations of the samples containing the SPIONs or proteins obtained under those conditions are shown in ESM, Figure S4.

### 2.3. Evaluation of the Optimized Method

During the subsequent experimental step, the method’s analytical performance was verified. For each analyte, the calibration curve was determined. Additionally, capillary recovery and inter- and intra-day repeatability parameters for peak area and migration time were calculated. Standard solutions of Fe_3_O_4_@COOH SPIONs (Fe concentrations ranging from 1 to 50 μg/mL), albumin (0.25 to 5 mg/mL), and transferrin (0.25 to 4 mg/mL) were analyzed under optimized conditions (ESM, Figures S5–S7). The linearity of the obtained curves was at a satisfactory level (R^2^ ≥ 0.995), the same as the recovery (≥80%) and the intra- (relative standard deviation; RSD ≤ 5%) and inter-day (RSD ≤ 8%) repeatability for the migration times (Table 3). Unfortunately, the unacceptable values were obtained in the case of the inter-day peak areas, proving the necessity of each-day calibration in the case of carrying out the quantitative analysis.

The possible explanation of the phenomenon can be related to at least a few aspects. Firstly, the interface between CE and ICP-MS/MS is a very delicate construction susceptible to various factors. After each experimental session, it is deconstructed due to the need to rinse individual elements and capillaries. Perfect reconstruction of the previous position during the next session may prove a challenge. Next, a nebulizer setup affects the draw rate of the sheath liquid and the flow rate of the sample, and thus the signal intensity for monitored masses. On the other hand, the inner surface of the capillary may undergo undesirable modifications by the compounds present in the samples or the BGE configurations during subsequent analyses affecting the complete migration of the reagents. An adverse effect of the analytes on the capillary was observed during the research on SPIONs and proteins. Despite the frequent rinsing, the signal quality, resolution, and capillary recoveries gradually deteriorated. For this reason, a regular capillary replacement was required (every 2 weeks). A nebulizing capillary can be clogged, even with systematic rinsing. Because of all these factors, the method may not be suitable for quantifying proteins and SPIONs in a routine manner unless the calibration curves are prepared during the same experimental session.

### 2.4. Investigation of Synthesized SPIONs

Before the SPION–protein interactions were investigated, first the method was used to scrutinize individual NPs and their stability during electrophoretic separation. Six different types of SPIONs were tested, commercially available (Fe_3_O_4_@COOH, Sigma-Aldrich, St. Louis, MO, USA) and synthesized: non-modified (Fe_3_O_4_), doped with Au (Fe_3_O_4_@Au), stabilized with polyethylene glycol (Fe_3_O_4_@PEG), polyethyleneimine (Fe_3_O_4_@PEI) or sodium citrate (Fe_3_O_4_@Citr). Initially, the research was conducted using nanoparticle cores obtained as described in the previous study [20]. However, due to their large size distribution, intense interactions with the capillary occurred, resulting in poor quality signals. At that moment, it was clear that more stringent conditions were needed during the SPIONs’ synthesis, and only these types of NPs were employed during the following experiments. Moreover, to have the possibility for brief comparison, the cores of the non-doped synthesized nanoparticles were obtained similarly, but the different stabilizers used for a surface modification determined the charge of the nanomaterial and their electrophoretic mobility. This resulted in a group of nanoparticles with identical superparamagnetic core morphology but substantially different surface characteristics. Table 4 shows the ζ-potential (ζ-zeta) of the synthesized SPIONs with different stabilizers. ζ-potential is the parameter that gives information about the charge of the nanoparticles and their tendency to formulate aggregates or remain discrete (regarding electrostatic stabilization). Based on the obtained values, Fe_3_O_4_@PEI and Fe_3_O_4_@Citr should be the most stable.

**Figure 1:**
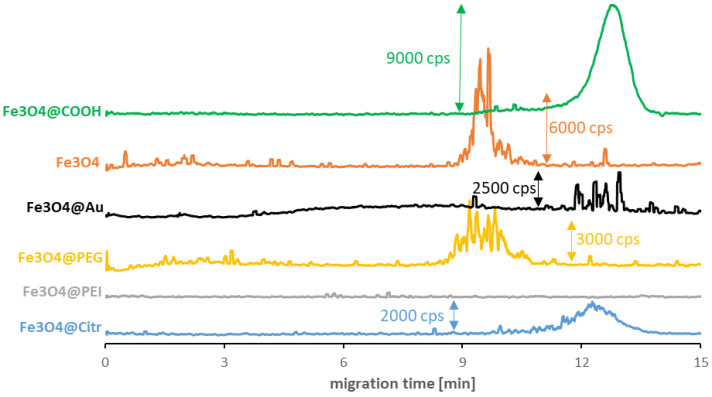
CE-ICP-MS/MS electropherograms of carboxyl-SPIONs (green) with 15 μg/mL Fe and synthesized SPIONs with 10–20 μg Fe/mL suspended in incubation buffer (10 mM phosphate buffer, pH 7.4, and 100 mM NaCl): non-modified (orange), doped with Au (black) stabilized with PEG (yellow), PEI (gray) and sodium citrate (blue); separation under optimized conditions (see Table 1 and Table 2), MS/MS signal ^56^Fe^16^O^+^. cps—counts per second.

As seen in Figure 1, surface modification is not the only factor determining the values of the SPIONs’ migration times. In some cases, it crosses out the possibility of their signal detection in a reasonable analysis time. The best quality signals among synthesized NPs were registered for Fe_3_O_4_@Citr. The strong signal disturbance for non-modified Fe_3_O_4_ and Fe_3_O_4_@PEG was caused by their agglomeration. Despite this phenomenon, the obtained results allow for establishing their average migration times. On the other hand, the lack of a distinctive signal in the case of Fe_3_O_4_@PEI suggests the presence of sufficiently strong interactions between the capillary inner surface (-SiO^−^ groups) and polyethyleneimine (-NH_3_^+^ groups), which resulted in the NPs remaining in the capillary during the measurement. It is worth mentioning that analysis of the commercially available Fe_3_O_4_@COOH SPIONs (with an amphiphilic polymer coating) resulted in the improved quality of the signal parameters. Still, the modification enormously reduced their magnetic properties, which can be a perfect explanation for the ease of their analysis and signal detection. Finally, the doping of nanoparticles with gold significantly impacted their structure, which resulted in both: the reduction in magnetic properties and the quality of the signal.

Synthesized SPIONs were also characterized—scanning-transmission electron microscopy (STEM) images for Fe_3_O_4_@Citr and Fe_3_O_4_@PEI were obtained (Figure S8). The diameter of the Fe_3_O_4_ nanoparticles in both cases does not exceed 10 nm, and we expect that other types of nanoparticles will have similar sizes. This can be explained by the fact that the entire family of nanoparticles was synthesized according to the well-known co-precipitation methodology [30,31]. Irrespective of the type of ligand, the same volume of ammonia was used in each case, ensuring the medium’s pH stability at the stage of precipitation of iron hydroxides and nucleation of nanostructures. Different types of ligands were added 10 min after the precipitation when the nanoparticles were already mainly formed, and the presence of the stabilizer had no significant effect on the diameter of the Fe_3_O_4_ cores obtained. In this case, the ligand only determines the nature of the surface and protects against aggregation.

### 2.5. Interactions of SPIONs with Albumin and Transferrin

**Figure 2:**
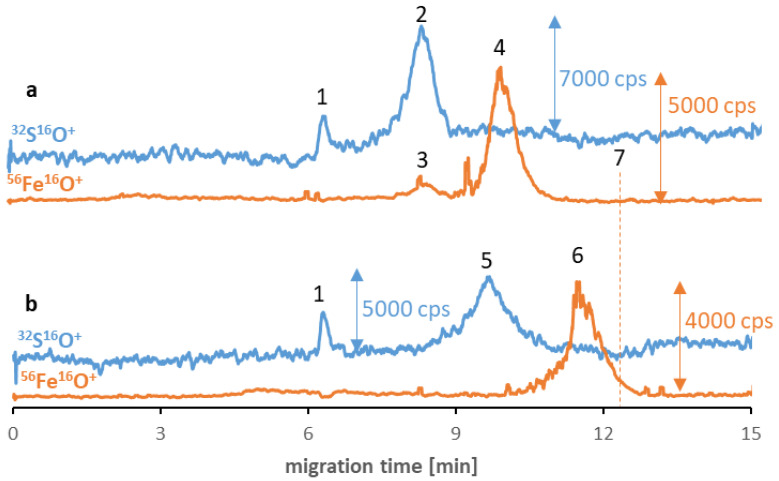
CE-ICP-MS/MS electropherograms’ MS/MS signals ^32^S^16^O^+^ and ^56^Fe^16^O^+^ of a mixture of Fe_3_O_4_@Citr (10–20 μg Fe/mL) and transferrin (**a**) and albumin (**b**), 1 mg/mL each, diluted in 10 mM phosphate buffer, pH 7.4, and 100 mM NaCl, and incubated at 37 °C for 6 h. Signal assignment: blank sulfur signal (1), transferrin (2), Fe from the transferrin (3), Fe_3_O_4_@Citr (4 and 6), albumin (5), Fe_3_O_4_@Citr analyzed individually (7); separation under optimized conditions (see Table 1 and Table 2).

Despite the low stability and polydispersity due to the agglomeration of the SPIONs in the buffer solutions, in some cases, the influence of proteinaceous media improves the parameters mentioned [20]. Consequently, in the last step of the examination, the applicability of the method for studying the SPIONs’ behavior in the proteinaceous media was examined. Two different proteins were chosen as potential reagents: albumin (the most abundant protein in human serum) and transferrin (the potential tumor-targeting ligand [32]). Two different samples were prepared for each type of NP, and their separation results were compared after six hours of incubation. Figure 2 shows Fe_3_O_4_@Citr SPIONs incubated with transferrin (a) and albumin (b). The obtained results suggest the absence of nano-protein conjugates as there is no visible overlap of sulfur and iron signals’ migration times, which can be assigned as proof for the protein corona formation. In the case of the transferrin sample, it was confirmed that a small peak (signal no. 3) corresponds to iron naturally present in the structure of this protein (25% of total transferrin is present in the bloodstream in the form of iron-saturated holo-transferrin) [33]. However, the differences in migration times of two signals of iron (no. 4 and 6) compared to the previously established time for Fe species (signal no. 7) and the visible correlation between the times of signals no. 4 and 6, and these corresponding to proteins (contain sulfur, no. 2 and 5) indicate the hypothesis of the possible interaction between reagents in both samples. The presence of proteins was notably the reason for the shift in the SPIONs’ migration times. As the eventual explanation for the correlation between the changes in the signal’s migration times, we look out for the protein corona formation in the sample and their breakdown during the separation of magnetic nanoobjects in the electric field. Similar to the surface modification, proteins that coat the NP’s surface determine the value of their migration times. The mentioned phenomenon is probably caused by the SPIONs’ interaction with the inner capillary wall due to their magnetism. To confirm that the change in migration time of the SPIONs was related to protein corona formation, the sample of Fe_3_O_4_@Citr incubated with albumin was subjected to ultrafiltration in order to remove the excess of free proteins. Then the >100 kDa fraction was subjected to analysis under optimized conditions. Despite the decrease in signal intensity (caused by the reduced recovery of conjugates from the filter), a similar observation (in comparison to the samples before ultrafiltration) was noted, confirming that the conjugates were present in the sample just before the electrophoretic analysis (ESM, Figure S9). Moreover, the lack of signals corresponding to the SPIONs in the filtrate (<100 kDa) was also noticed, suggesting that only free proteins passed through the filter.

**Figure 3:**
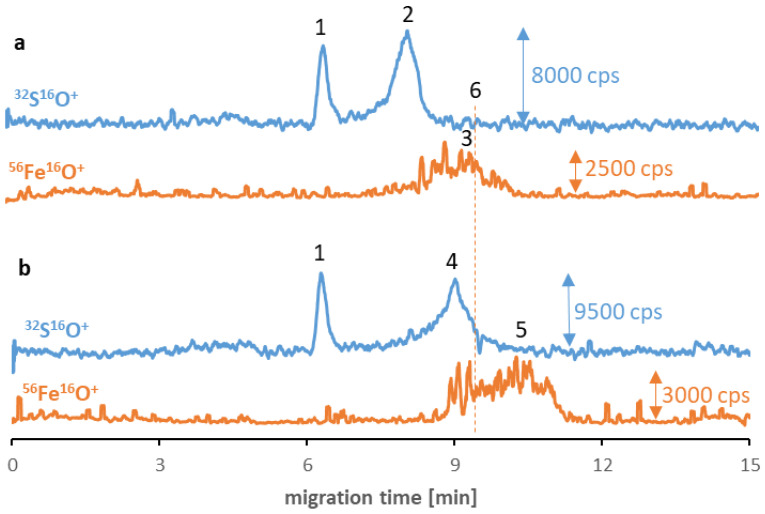
CE-ICP-MS/MS electropherograms’ MS/MS signals ^32^S^16^O^+^ and ^56^Fe^16^O^+^ of a mixture of Fe_3_O_4_@PEG (10–20 μg Fe/mL) and transferrin (**a**) and albumin (**b**), 1 mg/mL each, diluted in 10 mM phosphate buffer, pH 7.4, and 100 mM NaCl, and incubated at 37 °C for 6 h. Signal assignment: blank sulfur signal (1), transferrin (2), Fe_3_O_4_@PEG (3 and 5), albumin (4), Fe_3_O_4_@PEG analyzed individually (6); separation under optimized conditions (see Table 1 and Table 2).

Analogical tests were carried out for other synthesized SPIONs, and similar phenomena occurred in the case of Fe_3_O_4_@PEG (and non-modified Fe_3_O_4_, see ESM, Figure S10). As can be seen in Figure 3, the SPION signals (no. 3 and 5) have migration times longer than the transferrin (no. 2) and albumin (no. 4) signals and shifted to shorter times than before interaction with proteins. However, the signal quality for these types of SPIONs is worse due to their tendency to agglomerate. While PEG stabilization is primarily related to the strong steric effect (independent from the ζ-potential value and the charge of NPs), the agglomeration of Fe_3_O_4_@PEG may be caused by the vulnerability of the PEG coating to substitution due to the low affinity to the SPIONs. Concerning the Fe_3_O_4_@Au, despite the inferior quality of nanomaterial signals, incubation with albumin allowed for the stabilization of the nanomaterial. Moreover, analogous to previous analyses, the visible peak without disturbance appeared following the signal originating from the albumin (ESM, Figure S11). In the case of Fe_3_O_4_@PEI, no iron signals were detected on electropherograms despite incubating with proteins. Similar to other NPs, the reason can be attributed to the breakdown of the already formed protein corona during electrophoretic separation and, thus, the retention of SPIONs in the capillary. By contrast, in the case of Fe_3_O_4_@Au, the presence of albumin on their surface with a high possibility stabilized the nanomaterial structure, and the signal was noticed on the electropherogram.

Investigation of the SPIONs in more complex samples was also carried out. Fe_3_O_4_@Citr was incubated for 6 h with 10-times diluted human serum in order to study the behavior of synthesized nanoparticles in the presence of other matrix compounds. As albumin constitutes about 50–60% of the total plasma proteins, the obtained electropherogram (Figure S12) was similar to Figure 2b. Unfortunately, the examination of the interactions between SPIONs and other human serum proteins is laborious in real samples due to the still insufficient resolution between many compounds and, on top of that, a high concentration of albumin (38–50 g/L in relation to, e.g., 2–3 g/L of transferrin), which can obscure other signals.

Considering previous research [29], in which the protein corona formation was confirmed based on the overlap of albumin and nanoparticle signals, the question arises—what is the reason for the difference in the obtained results between the commercially available and self-synthesized NPs? The answer can be strictly related to the different magnetism of the investigated nanomaterials (strong for synthesized, poor in the case of the custom product), as, undoubtedly shown above, its presence strongly affects the processes taking place in the separation capillary. In summary, the purchased SPIONs displayed poor magnetic properties, so their migration in the capillary during electrophoretic separation was predictable, and the biologically occurred forms were stable. In contrast, currently used SPIONs exhibit remarkable magnetic properties, and thus, their behaviors in the capillary after applying the voltage resulted in the decomposition of nano–bio forms or their in-capillary further changes. On the other side, the mentioned issues can be treated as a premise that strongly superparamagnetic nanomaterials are not able to form durable interactions (bonds?) with proteins. This observation shed new light on the necessity of changing the procedures of protein corona discerning investigation.

## 3. Materials and Methods

### 3.1. Chemicals

All reagents used throughout this study were at least of analytical grade. Iron(II) chloride tetrahydrate, iron(III) chloride hexahydrate, polyethyleneimine (branched, M_w_ = 25,000 Da), sodium chloride, sodium hydroxide, sodium hydrogen phosphate, sodium dihydrogen phosphate, and gold(III) chloride trihydrate were obtained from Sigma-Aldrich (St. Louis, MO, USA). The 25% ammonium hydroxide was the product of Chempur (Piekary Śląskie, Poland). Tris hydrochloride (tris(hydroxymethyl)aminomethane hydrochloride), sodium citrate dihydrate, iron, and sulfur ICP standard solutions were purchased from Merck Millipore (Darmstadt, Germany). Vanadium standard solution was obtained from Fluka (Buchs, Switzerland). Poly(ethylene glycol) (M_w_ = 800 Da) was the product of Thermo Scientific (Geel, Belgium). Albumin and transferrin from human serum and superparamagnetic iron oxide nanoparticles with an amphiphilic polymer coating terminated with carboxyl groups (25 nm core size, Fe_3_O_4_@COOH) were obtained from Sigma-Aldrich (St. Louis, MO, USA). Methanol (LC-MS grade) was the product of POCh (Gliwice, Poland). The nitrogen of purity ≥99.999% applied during SPIONs’ synthesis and oxygen of purity ≥99.999% used for the collision/reaction cell in ICP-MS/MS were purchased from Messer (Bad Soden, Germany). Ultrapure Milli-Q water was obtained from a Millipore Elix 3 system (Merck Millipore) and used throughout this study.

### 3.2. SPIONs Synthesis

The reaction was carried out at room temperature, under an inert gas (nitrogen) atmosphere, with constant stirring at a speed of 2000 rpm. In a 250 mL three-neck flask, 4.886 g FeCl_3_ ∙ 6 H_2_O and 2.982 g FeCl_2_ ∙ 4 H_2_O were dissolved in 120 mL water. The solution was deoxygenated by nitrogen purging for 10 min and heated to 90 °C. Then, after the iron salt had dissolved entirely, 15 mL of a 25% aqueous ammonia solution was quickly injected into the flask. After another 10 min, 20 mL of one of the selected stabilizers was added as an aqueous solution. Three different stabilizers were used: branched polyethyleneimine (20 mg/mL), polyethylene glycol (20 mg/mL), and sodium citrate (20 mg/mL). In synthesizing non-stabilized nanoparticles, the above-described step was omitted. The process of stirring and heating in an inert gas atmosphere was carried out for 2 h. After the end of the synthesis, the cooled nanoparticle suspension was purified six times by magnetic decantation and washed with ultrapure water. In the case of Au-doped nanoparticles, the reaction was carried out under similar conditions but on a different scale. Namely, 488.6 mg FeCl_3_ ∙ 6 H_2_O and 298.2 mg FeCl_2_ ∙ 4 H_2_O were dissolved in 120 mL of water. In parallel, a mixture of NaOH and HAuCl_4_ was prepared by adding 3.3 mL of 100 mM aqueous HAuCl_4_ to 7.5 mL of 1M NaOH. The molar ratio of Fe to Au precursors was 10:1. Then, when the iron salt was completely dissolved, the mixture of NaOH and HAuCl_4_ was quickly added to the flask and stirred continuously at 90 °C under a nitrogen atmosphere for 2 h. After the synthesis was completed, the nanoparticle suspension was cleaned three times and separated from the gold nanoparticles formed in parallel by magnetic decanting. All finished NPs were suspended in ultrapure water. The presence of Au in the doped nanoparticles was confirmed by optical emission spectrometry (AvaSpec AVS-Desktop-USB2, Avantes, NS Appledoorn, The Netherlands) [34].

### 3.3. Sample Preparation

All samples were prepared in an incubation buffer simulating the physiological environment (10 mM phosphate buffer containing 100 mM NaCl, pH 7.4). Commercially available SPIONs were diluted to 15 μg/mL Fe. Synthesized nanoparticles were subjected to brief vortexing (MX-S Vortex, Scilogex, Rocky Hill, CT, USA) before they were 1000 times diluted (final Fe concentration in the range from 10 to 20 µg/mL). Both albumin and transferrin samples were diluted to 1 mg/mL. Human serum protein–SPION samples were prepared by adding NP solution to an individual protein. Samples were incubated at a temperature of 37 °C and stirred at 400 rpm for desired time using a MultiTherm incubator (Benchmark, Lodi, NJ, USA). The Fe_3_O_4_@Citr–albumin sample was subjected to ultrafiltration through a 100 kDa filter (Amicon Ultra 0.5 mL) at 6000 rcf for 40 min in normal and then reversed mode using MPW-352RH centrifuge (MPW Med. Instruments, Warsaw, Poland).

### 3.4. CE-ICP-MS/MS Instrumentation

Analyses were conducted on the CE-ICP-MS/MS hyphenation: an Agilent 7100 CE system and an Agilent 8900 ICP tandem mass spectrometer (Agilent Technologies, Waldbronn, Germany) working in MS/MS mode using collision/reaction cell. A CEI-100 nebulizer interface (Teledyne CETAC Technologies, Omaha, NE, USA) equipped with a low-volume spray chamber and a cross-piece merging the sheath liquid flow (containing 10 ppb ^51^V in 10-times diluted BGE) was used for the liquid introduction system. The CE electrical circuit was closed by the platinum wire and maintained by the constant flow of the sheath liquid.

Polyimide-coated fused silica capillaries (i.d. 75 μm, o.d. 375 μm, length 70 cm) were obtained from C&M Scientific Ltd. (Silsden, UK). Newly used capillaries were activated by rinsing with 1 M NaOH for 40 min, a washing mixture of 1 M NaOH, methanol, and water (1/2/1, *v*/*v*/*v*) for 40 min, and purged with water for 20 min. Additionally, the capillary was conditioned with 1 M NaOH (10 min), washing mixture (5 min), water (5 min), and BGE (10 min) at the beginning of each experimental session. Between the analyses, it was rinsed with 1 M NaOH (2 × 0.5 min), washing mixture (0.5 min), water (0.5 min), and BGE (2 × 0.5 min). Moreover, the nebulizer was rinsed with methanol (5 min), water (5 min), and methanol (5 min) at the beginning and the end of each experimental session.

Vanadium, used as an internal standard in sheath liquid, allowed for observation of the hyphenation stability and nebulization efficiency during analyses. Those were initiated when the ^51^V^16^O^+^ signal was sufficiently high (counts per second, cps, >4000) and stable (relative standard deviation, RSD, >2%).

Agilent MassHunter 4.5 Workstation (version C.01.06) and Agilent OpenLab ChemStation (version C.01.09) software were used for the instrument control and data analysis (accessed on 8 July 2022).

### 3.5. ζ-Potential Measurement

ζ-potential measurements of synthesized SPIONs were carried out in disposable polystyrene cuvettes using a Zetasizer Nano ZS device (Malvern Panalytical, Malvern, UK) equipped with a dip cell with palladium electrodes (Malvern). Suspensions of different SPIONs were diluted with water to obtain the appropriate optical density for ζ-potential measurements and then briefly (~10 s) sonicated (ultrasonic cleaner Sonic-5, Polsonic, Warsaw, Poland) before the analysis. All measurements were conducted at 25 °C after 60 s of temperature stabilization and replicated three times per each type of NPs.

### 3.6. Morphology Characterization of Synthesized SPIONs

Scanning transmission electron microscopy (STEM) micrographs for Fe_3_O_4_@Citr and Fe_3_O_4_@PEI nanoparticles were captured at 30.0 kV accelerating voltage by means of Hitachi SU8230 ultra-high-resolution field emission scanning-transmission electron microscope (Hitachi High-Technologies Corporation, Tokyo, Japan) using copper TEM grids coated with Lacey carbon film.

## 4. Conclusions

To summarize, many problems and red flags emerged during the application of CE-ICP-MS/MS for studying SPION–human serum protein transformations. Firstly, the high resolution between SPIONs and protein signals is required for the method to be applicable for studying those analytes. While analysis of individual reagents is usually possible, samples containing self-synthesized strongly magnetic SPIONs incubated with serum proteins are highly challenging. Next, the optimal operational parameters need to be chosen as the compromise between two such different compounds, and because of the variety of surface modifications, it is not easy to prepare one versatile method that could be used to study samples consisting of different SPIONs. On top of that, the protein corona’s lack of durability under electrophoretic separation conditions is a significant limitation in the investigation of NP changes.

The study concludes that, at this research stage, we are standing at a crossroads and divagate how we can achieve the robust speciation analysis of such fastidious analyte as conjugates of synthesized SPIONs with proteins burdened with high spectral interferences and for which applying the high-resolution separation technique as capillary electrophoresis is not enough. Moreover, while the magnetic properties are sought due to the potential applications of SPIONs, they are also a reason for the difficulties during their reliable analysis. The solution to the mentioned issues can be addressed by the selective modification of the inner capillary walls and by outlining our future tasks.

## Figures and Tables

**Table 1:** ICP-MS/MS optimized operational parameters.

Parameter	Setting
RF power	1570 W
Sample Depth	8.40 mm
Torch Width	1.5 mm
Nebulizer (Ar) gas flow	0.90 L/min
Reaction (O_2_) gas flow	30%
Sampler and skimmer cones	Pt
Monitored masses	^51^V^16^O^+^, ^32^S^16^O^+^, ^56^Fe^16^O^+^

**Table 2:** CE optimized operational parameters.

Parameter	Setting
Background electrolyte	Tris hydrochloride 5 mM, pH 7.4
Temperature	37 °C
Voltage	+15 kV
Current	6–7 µA
Sample injection	20 mbar × 5 s
Capillary	Polyimide-coated fused silica capillary, i.d. ^1^ 75 μm, o.d. ^2^ 375 μm, length 70 cm

^1^ i.d.—the internal diameter. ^2^ o.d.—the outer diameter.

**Table 3:** Figures of merit of the optimized CE-ICP-MS/MS method.

Analyte	Linearity, R^2^	Capillary Recovery (%) (*n* = 3)	RSD (%)			
			Migration time		Peak area	
			Intraday (*n* = 3)	Inter-day (*n* = 3)	Intraday (*n* = 3)	Inter-day (*n* = 3)
Albumin	0.9968	88.61	0.25	5.17	7.52	15.54
Transferrin	0.9982	89.62	2.14	5.28	7.99	16.18
SPIONs	0.9996	89.31	1.02	5.84	4.71	13.35

**Table 4:** ζ-Potential of synthesized SPIONs (*n* = 3).

SPIONs	ζ-Potential
Fe_3_O_4_	24.8 ± 0.2
Fe_3_O_4_@PEG	−3.7 ± 0.5
Fe_3_O_4_@PEI	48.6 ± 0.5
Fe_3_O_4_@Citr	−29.0 ± 1.8

## Data Availability

The data presented in this study are available in supplementary material.

## Supplementary Materials

The following supporting information can be downloaded at: https://www.mdpi.com/article/10.3390/molecules27238442/s1. Figure S1:Evaluation of the oxygen flow rate in collision/reaction cell based on the S/N and signal intensity for ^56^Fe^16^O^+^ (a) and ^32^S^16^O^+^ (b) signals. The evaluation was based on the solution containing iron standard (50 ng/mL) and sulfur standard (250 ng/mL) in 2% HNO_3_; Figure S2: Effect of the injection volume on the resolution of albumin (1 mg/mL), transferrin (1 mg/mL), and carboxyl-SPIONs (15 μg Fe/mL) for the samples of proteins and SPIONs measured independently. BGE: tris hydrochloride 5 mM, pH 7.4, voltage: +18 kV, MS/MS signals: ^56^Fe^16^O^+^ and ^32^S^16^O^+^, *n* = 3; Figure S3: Effect of the applied voltage on the resolution of albumin (1 mg/mL), transferrin (1 mg/mL) and carboxyl-SPIONs (15 μg Fe/mL) for the samples of proteins and SPIONs measured independently. BGE: tris hydrochloride 5 mM, pH 7.4, injection volume: 100 mbar × s, MS/MS signals: ^56^Fe^16^O^+^ and ^32^S^16^O^+^, *n* = 3; Figure S4: CE-ICP-MS/MS electropherograms’ of (A) albumin and transferrin (1 mg/mL, ^32^S^16^O^+^ signal), and (B) carboxyl-SPIONs (15 μg Fe/mL, ^56^Fe^16^O^+^ signal). Signal assignments: blank sulfur signal (1), transferrin (2), albumin (3), carboxyl-SPIONs (4); separation under optimized conditions (Table 1 and Table 2); Figure S5: The linear range of the optimized CE-ICP-MS/MS method, MS/MS signal ^32^S^16^O^+^, albumin diluted in 10 mM phosphate buffer, pH 7.4, and 100 mM NaCl, *n* = 3; Figure S6: The linear range of the optimized CE-ICP-MS/MS method, MS/MS signal ^32^S^16^O^+^, transferrin diluted in 10 mM phosphate buffer, pH 7.4, and 100 mM NaCl, *n* = 3; Figure S7: The linear range of the optimized CE-ICP-MS/MS method, MS/MS signal ^56^Fe^16^O^+^, Fe_3_O_4_@COOH diluted in 10 mM phosphate buffer, pH 7.4, and 100 mM NaCl, *n* = 3; Figure S8: Fragments of STEM images of synthesized SPIONs: Fe_3_O_4_@PEI (a) and Fe_3_O_4_@Citr (b); Figure S9: CE-ICP-MS/MS electropherograms’ MS/MS signals ^32^S^16^O^+^ and ^56^Fe^16^O^+^ of mixture of Fe_3_O_4_@Citr (10–20 μg Fe/mL) and 1 mg/mL albumin, diluted in 10 mM phosphate buffer, pH 7.4, and 100 mM NaCl, incubated at 37 °C for 6 h and subjected to ultrafiltration. Signal assignment: blank sulfur signal (1), albumin (2), Fe_3_O_4_@Citr (3); separation under optimized conditions (see Table 1 and Table 2); Figure S10: CE-ICP-MS/MS electropherograms’ MS/MS signals ^32^S^16^O^+^ and ^56^Fe^16^O^+^ of mixture of Fe_3_O_4_ (10–20 μg Fe/mL) and transferrin (**a**) and albumin (**b**), 1 mg/mL each, diluted in 10 mM phosphate buffer, pH 7.4, and 100 mM NaCl, and incubated at 37 °C for 24 h. Signal assignment: blank sulfur signal (1), transferrin (2), Fe from the transferrin (3), Fe_3_O_4_ (4), albumin (5), Fe_3_O_4_ analyzed individually (6); separation under optimized conditions (see Table 1 and Table 2); Figure S11: CE-ICP-MS/MS electropherograms’ MS/MS signals ^32^S^16^O^+^ and ^56^Fe^16^O^+^ of mixture of Fe_3_O_4_@Au (10–20 μg Fe/mL) and transferrin (**a**) and albumin (**b**), 1 mg/mL each, diluted in 10 mM phosphate buffer, pH 7.4, and 100 mM NaCl, and incubated at 37 °C for 24 h. Signal assignment: blank sulfur signal (1), transferrin (2), Fe from the transferrin (3), Fe_3_O_4_@Au (4 and 7), Fe_3_O_4_@Au analyzed individually (5), albumin (6),; separation under optimized conditions (see Table 1 and Table 2); Figure S12: CE-ICP-MS/MS electropherograms’ MS/MS signals ^32^S^16^O^+^ and ^56^Fe^16^O^+^ of mixture of Fe3O4@Citr (10–20 μg Fe/mL) and human serum, diluted by 10 times with 10 mM phosphate buffer, pH 7.4, and 100 mM NaCl, and incubated at 37 °C for 6 h. Signal assignment: blank sulfur signal (1), albumin (2), Fe_3_O_4_@Citr (3), Fe_3_O_4_@Citr analyzed individually (4); separation under optimized conditions (see Table 1 and Table 2).
