# Supplementary material for: Red Flags and Adversities on the Way to the Robust CE-ICP-MS/MS Quantitative Monitoring of Self-Synthesized Magnetic Iron Oxide(II, III)-Based Nanoparticle Interactions with Human Serum Proteins
Source: Molecules. 2022 Dec 2;27(23):8442. doi: 10.3390/molecules27238442 (PMC9739417; doi:10.3390/molecules27238442)
Supplement: Supplementary file 1 [file molecules-27-08442-s001.zip › molecules-2050372-supplementary.pdf]

Molecules

Electronic Supplementary Material

Red flags and adversities on the way to the robust CE-ICP-MS/MS quantitative monitoring of self-synthesized magnetic iron oxide(II, III)-based nanoparticle interactions with human serum proteins

Jacek Sikorski, Marcin Drozd, Magdalena Matczuk

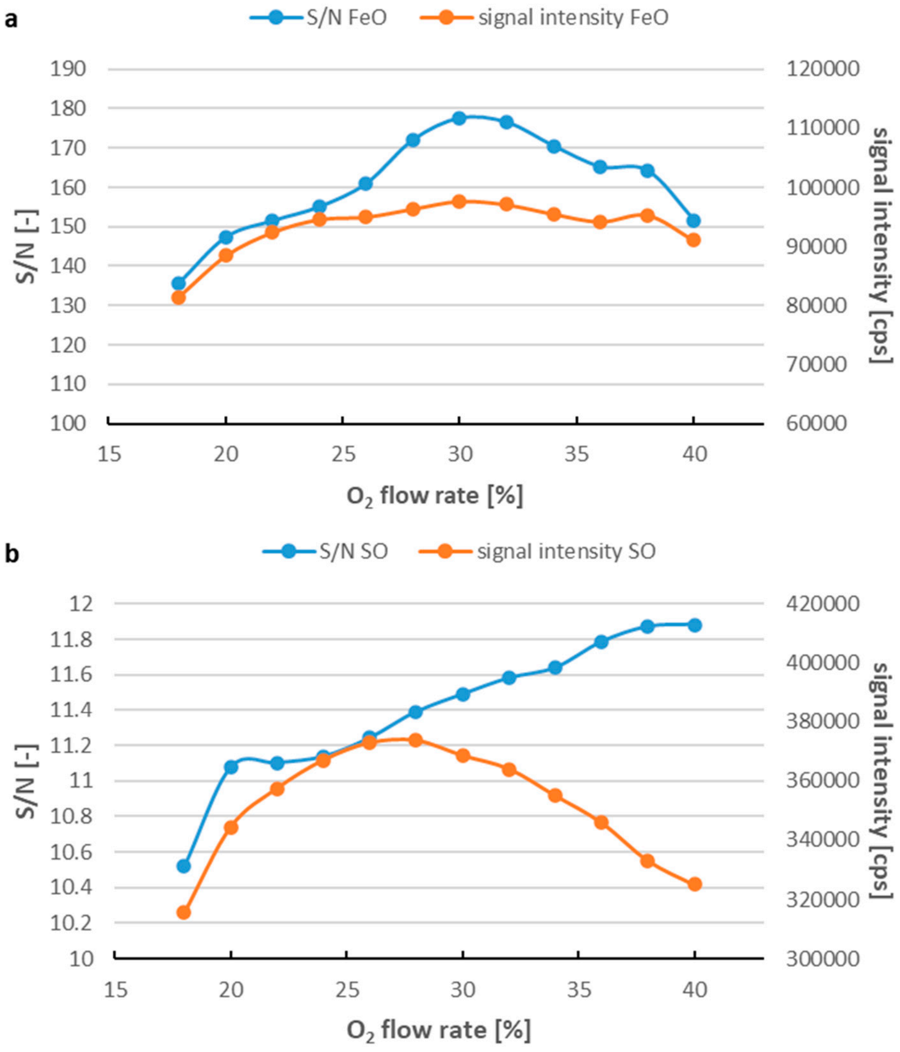

Figure S1. Evaluation of the oxygen flow rate in collision/reaction cell based on the S/N and signal intensity for $^{56}Fe^{16}O^{+}$ (**a**) and $^{32}S^{16}O^{+}$ (**b**) signals. The evaluation was based on the solution containing iron standard (50 ng/mL) and sulfur standard (250 ng/mL) in 2% HNO_3.

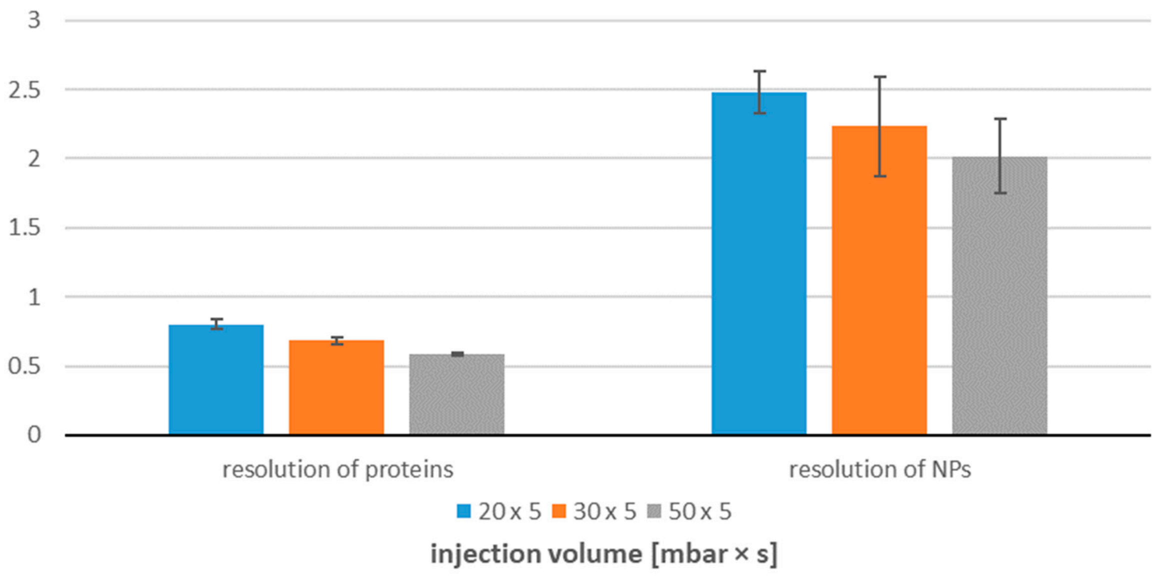

Figure S2. Effect of the injection volume on the resolution of albumin (1 mg/mL), transferrin (1 mg/mL), and carboxyl-SPIONs (15 µg Fe/mL) for the samples of proteins and SPIONs measured independently. BGE: tris hydrochloride 5 mM, pH 7.4, voltage: +18 kV, MS/MS signals: $^{56}Fe^{16}O^{+}$ and $^{32}S^{16}O^{+}$, n=3.

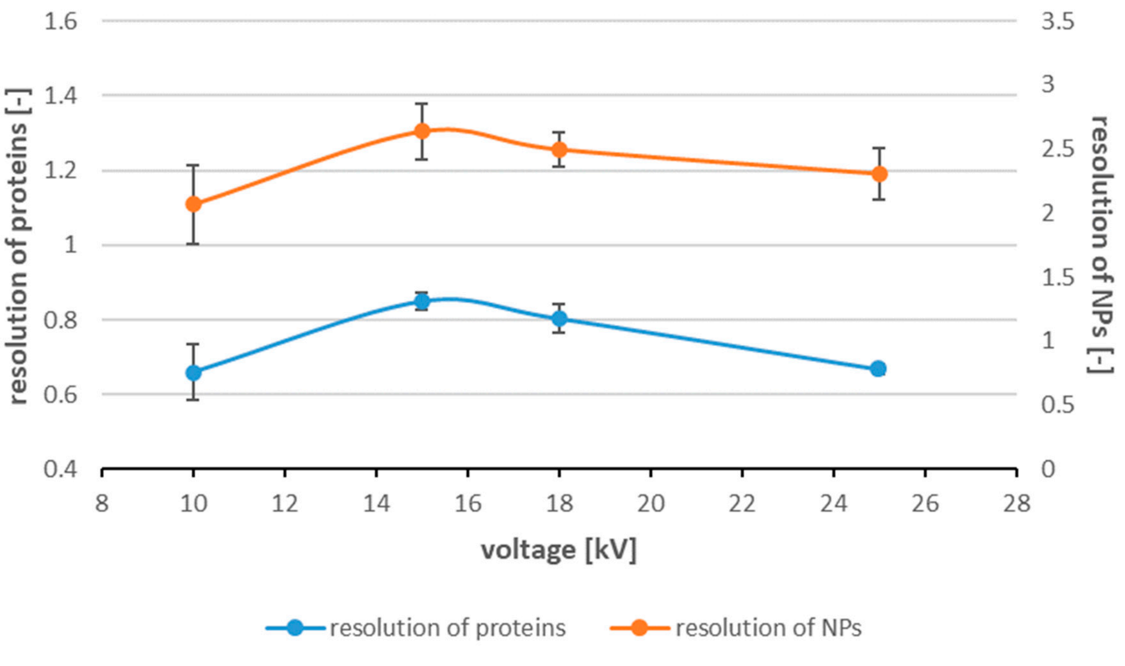

Figure S3. Effect of the applied voltage on the resolution of albumin (1 mg/mL), transferrin (1 mg/mL) and carboxyl-SPIONs (15 µg Fe/mL) for the samples of proteins and SPIONs measured independently. BGE: tris hydrochloride 5 mM, pH 7.4, injection volume: 100 mbar × s, MS/MS signals: $^{56}Fe^{16}O^{+}$ and $^{32}S^{16}O^{+}$, n=3.

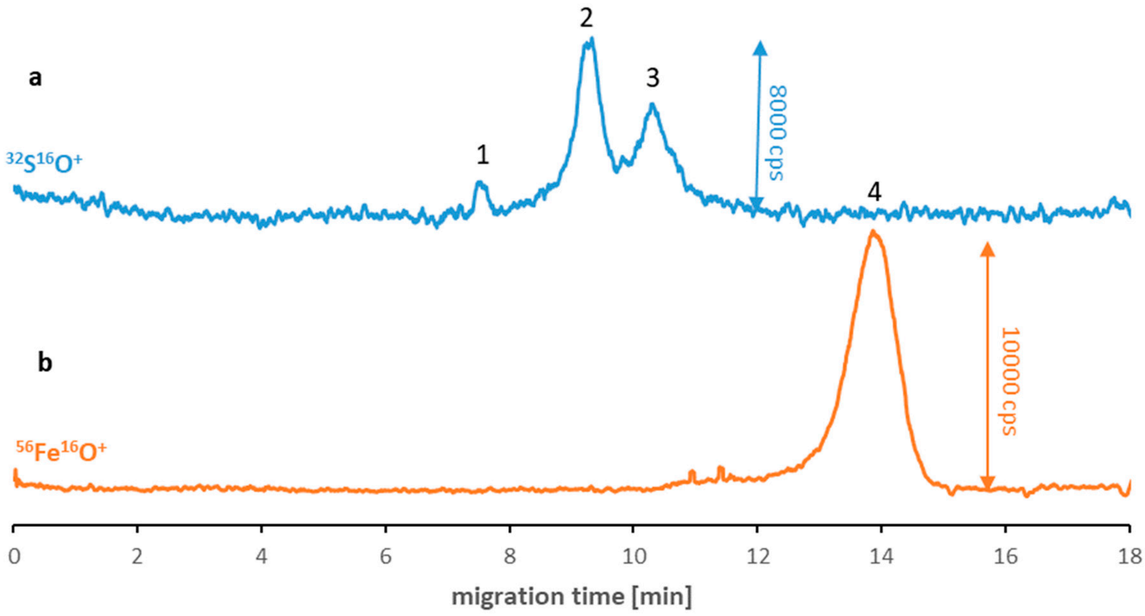

Figure S4. CE-ICP-MS/MS electropherograms' of (A) albumin and transferrin (1 mg/mL, $^{32}S^{16}O^+$ signal), and (B) carboxyl-SPIONs (15 µg Fe/mL, $^{56}Fe^{16}O^+$ signal). Signal assignments: blank sulfur signal (1), transferrin (2), albumin (3), carboxyl-SPIONs (4); separation under optimized conditions (Table 1 and 2).

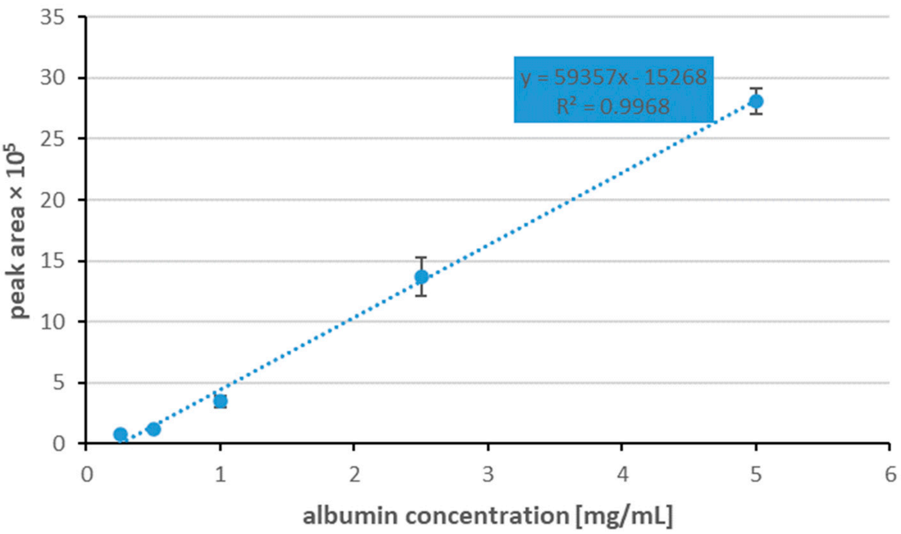

Figure S5. The linear range of the optimized CE-ICP-MS/MS method, MS/MS signal $^{32}S^{16}O^+$, albumin diluted in 10 mM phosphate buffer, pH 7.4, and 100 mM NaCl, n=3.

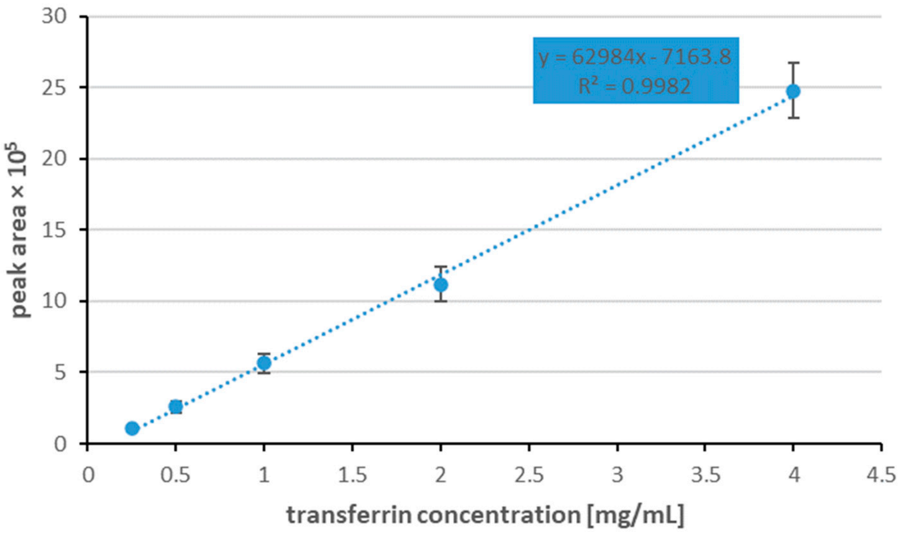

Figure S6. The linear range of the optimized CE-ICP-MS/MS method, MS/MS signal $^{32}S^{16}O^+$, transferrin diluted in 10 mM phosphate buffer, pH 7.4, and 100 mM NaCl, n=3.

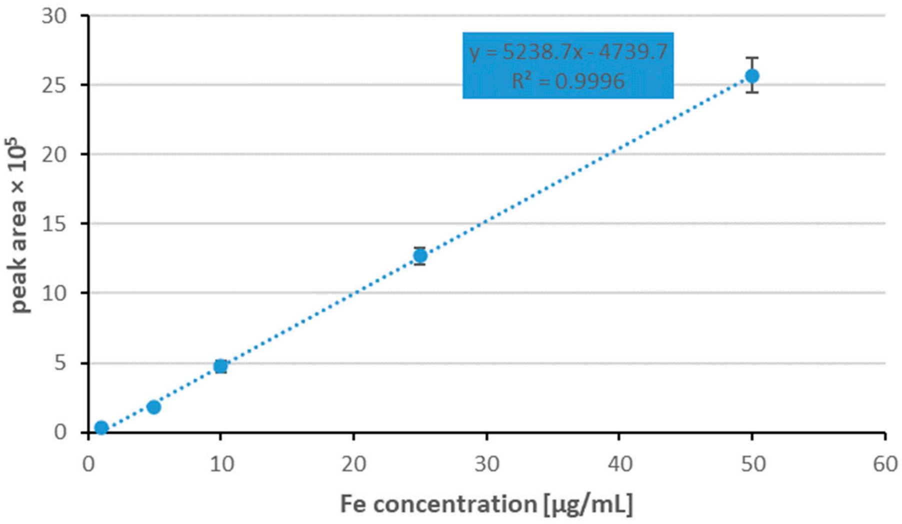

Figure S7. The linear range of the optimized CE-ICP-MS/MS method, MS/MS signal $^{56}Fe^{16}O^+$, Fe_3O_4@COOH diluted in 10 mM phosphate buffer, pH 7.4, and 100 mM NaCl, n=3.

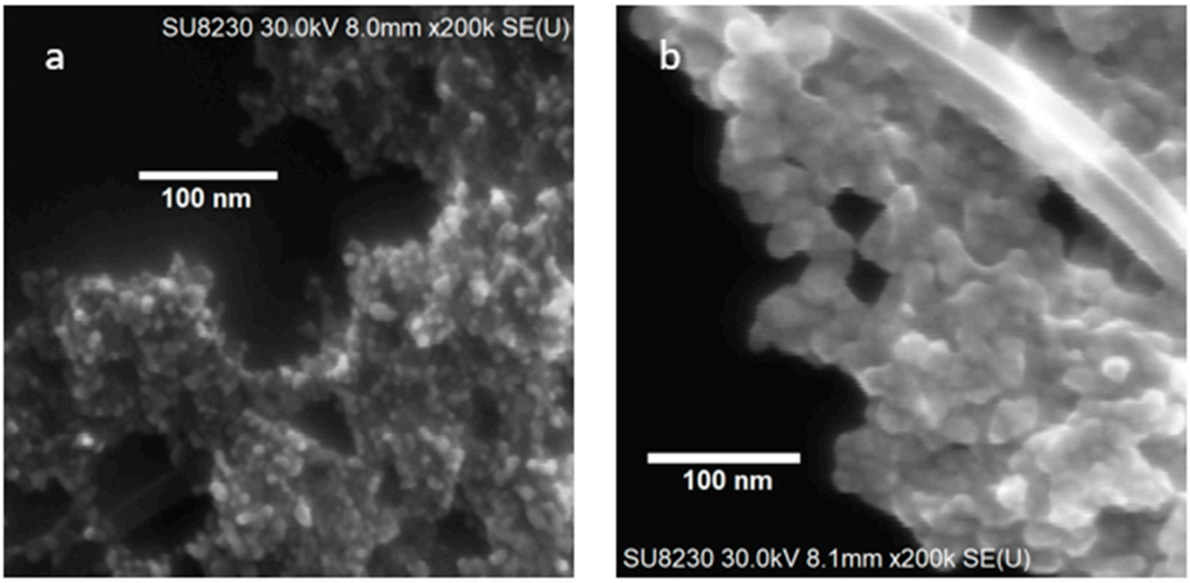

Figure S8. Fragments of STEM images of synthesized SPIONs: Fe_3O_4@PEI (a) and Fe_3O_4@Citr (b).

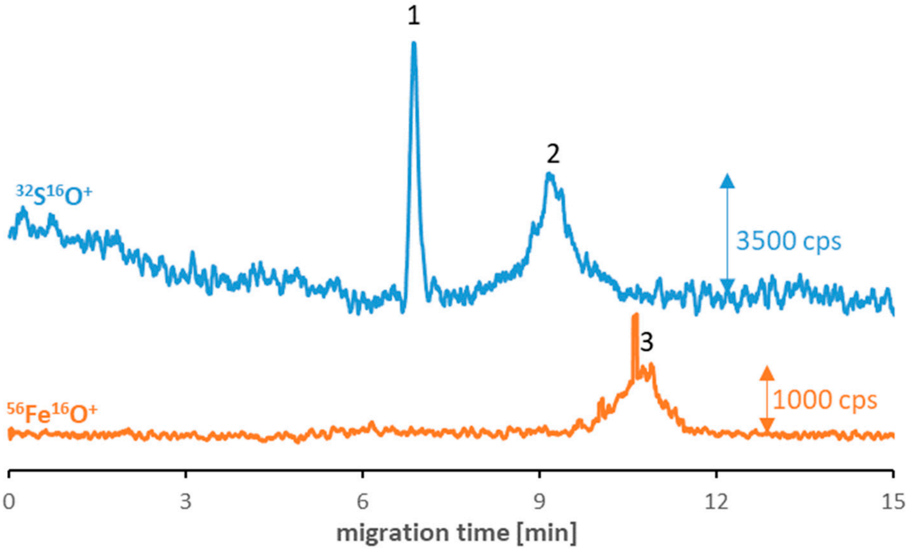

Figure S9. CE-ICP-MS/MS electropherograms' MS/MS signals $^{32}S^{16}O^+$ and $^{56}Fe^{16}O^+$ of mixture of Fe_3O_4@Citr (10-20 µg Fe/mL) and 1 mg/mL albumin, diluted in 10 mM phosphate buffer, pH 7.4, and 100 mM NaCl, incubated at 37 °C for 6 h and subjected to ultrafiltration. Signal assignment: blank sulfur signal (1), albumin (2), Fe_3O_4@Citr (3); separation under optimized conditions (see Table 1 and 2).

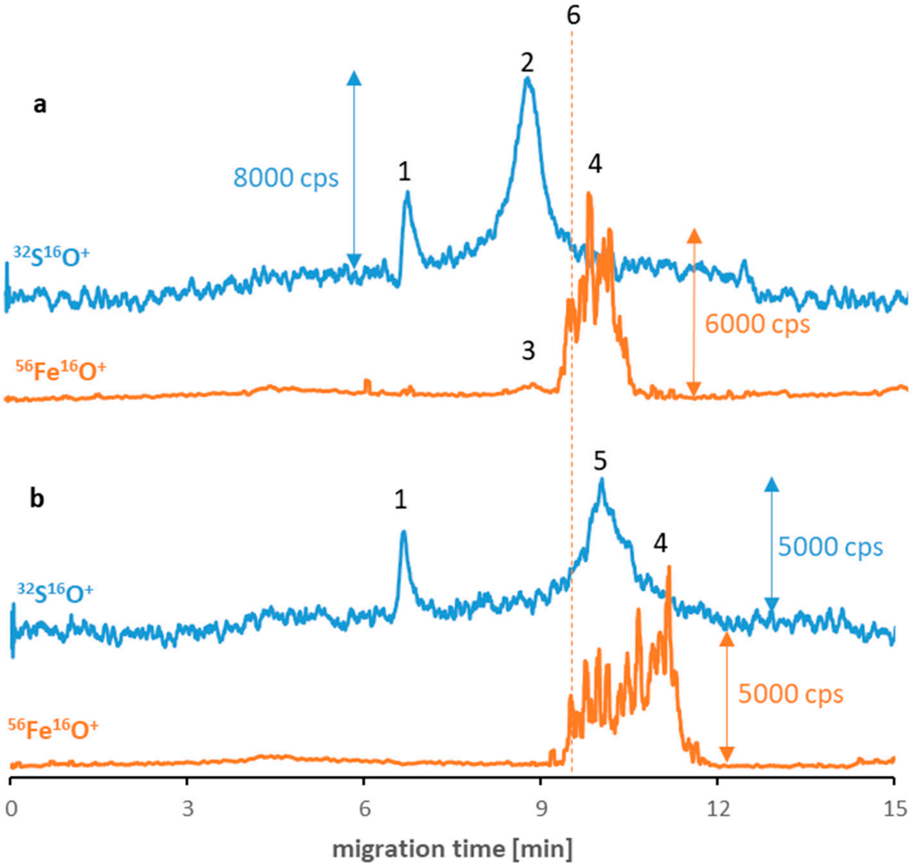

Figure S10. CE-ICP-MS/MS electropherograms' MS/MS signals $^{32}S^{16}O^+$ and $^{56}Fe^{16}O^+$ of mixture of Fe_3O_4 (10-20 µg Fe/mL) and transferrin (**a**) and albumin (**b**), 1 mg/mL each, diluted in 10 mM phosphate buffer, pH 7.4, and 100 mM NaCl, and incubated at 37 °C for 24 h. Signal assignment: blank sulfur signal (1), transferrin (2), Fe from the transferrin (3), Fe_3O_4 (4), albumin (5), Fe_3O_4 analyzed individually (6); separation under optimized conditions (see Table 1 and 2).

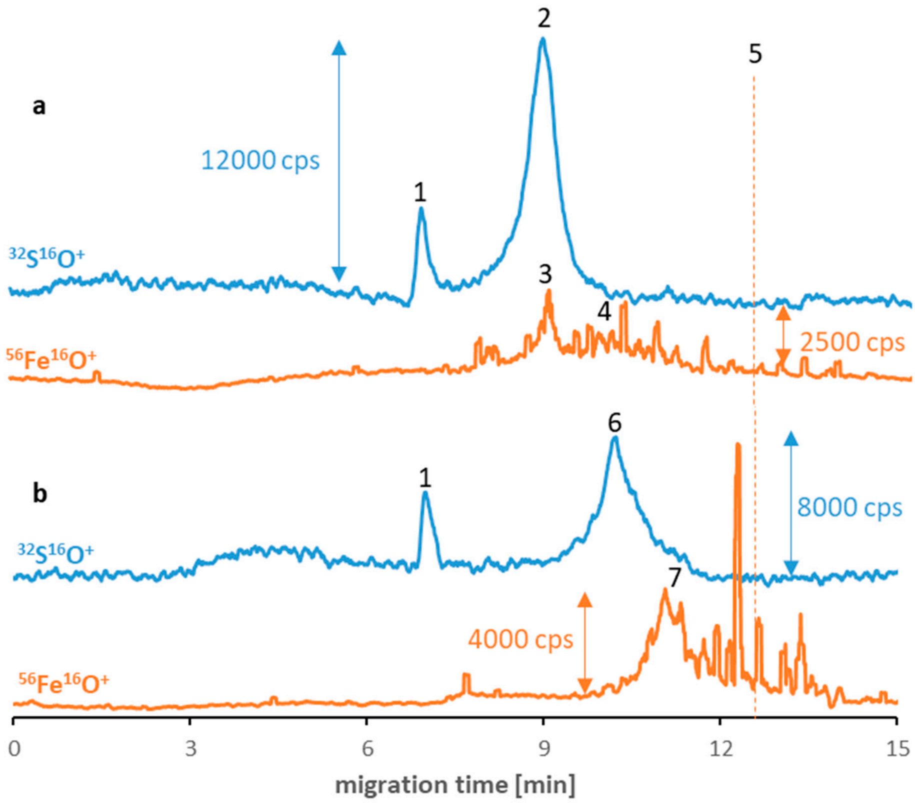

Figure S11. CE-ICP-MS/MS electropherograms' MS/MS signals $^{32}S^{16}O^+$ and $^{56}Fe^{16}O^+$ of mixture of Fe_3O_4@Au (10-20 µg Fe/mL) and transferrin (**a**) and albumin (**b**), 1 mg/mL each, diluted in 10 mM phosphate buffer, pH 7.4, and 100 mM NaCl, and incubated at 37 °C for 24 h. Signal assignment: blank sulfur signal (1), transferrin (2), Fe from the transferrin (3), Fe_3O_4@Au (4 and 7), Fe_3O_4@Au analyzed individually (5), albumin (6),; separation under optimized conditions (see Table 1 and 2).

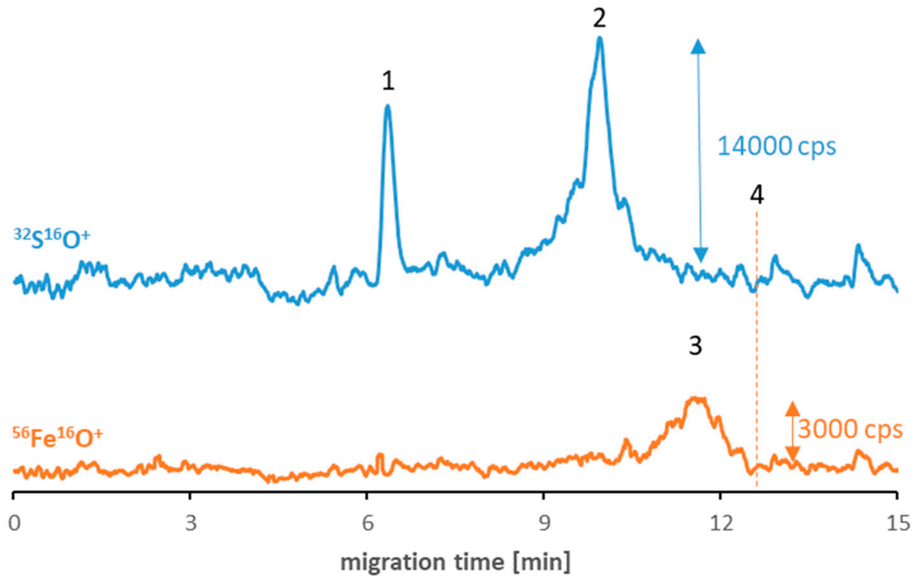

Figure S12. CE-ICP-MS/MS electropherograms' MS/MS signals $^{32}S^{16}O^+$ and $^{56}Fe^{16}O^+$ of mixture of Fe3O4@Citr (10-20 µg Fe/mL) and human serum, diluted by 10 times with 10 mM phosphate buffer, pH 7.4, and 100 mM NaCl, and incubated at 37 °C for 6 h. Signal assignment: blank sulfur signal (1), albumin (2), Fe_3O_4@Citr (3), Fe_3O_4@Citr analyzed individually (4); separation under optimized conditions (see Table 1 and 2).
